# Inhibition of ASIC1a Improves Behavioral Recovery after Stroke

**DOI:** 10.1523/ENEURO.0341-23.2023

**Published:** 2024-02-09

**Authors:** Ariel Armstrong, Tao Yang, Tiandong Leng, Zhi-Gang Xiong

**Affiliations:** Department of Neurobiology, Neuroscience Institute, Morehouse School of Medicine, Atlanta, Georgia 30310

**Keywords:** behavior, ischemia, recovery, stroke

## Abstract

Stroke continues to be a leading cause of death and long-term disabilities worldwide, despite extensive research efforts. The failure of multiple clinical trials raises the need for continued study of brain injury mechanisms and novel therapeutic strategies for ischemic stroke. The contribution of acid-sensing ion channel 1a (ASIC1a) to neuronal injury during the acute phase of stroke has been well studied; however, the long-term impact of ASIC1a inhibition on stroke recovery has not been established. The present study sought to bridge part of the translational gap by focusing on long-term behavioral recovery after a 30 min stroke in mice that had ASIC1a knocked out or inhibited by PcTX1. The neurological consequences of stroke in mice were evaluated before and after the stroke using neurological deficit score, open field, and corner turn test over a 28 d period. ASIC1a knock-out and inhibited mice showed improved neurological scores more quickly than wild-type control and vehicle-injected mice after the stroke. ASIC1a knock-out mice also recovered from mobility deficits in the open field test more quickly than wild-type mice, while PcTX1-injected mice did not experience significant mobility deficits at all after the stroke. In contrast to vehicle-injected mice that showed clear-sidedness bias in the corner turn test after stroke, PcTX1-injected mice never experienced significant-sidedness bias at all. This study supports and extends previous work demonstrating ASIC1a as a potential therapeutic target for the treatment of ischemic stroke.

## Significance Statement

The contribution of acid-sensing ion channels to neuronal injury is well studied; however, most work focuses on acute-phase molecular and histological endpoints. Here, we begin to bridge the bench-to-bedside translational gap by using clinically relevant endpoints in our preclinical model. We show for the first time that both genetic knock-out and acute pharmacological inhibition of ASIC1a improve long-term behavioral recovery after stroke. By performing neurological and behavioral tests over 28 d, we found that deletion or inhibition of ASIC1a resulted in faster recovery of focal and general neurological deficits and mobility. This work is an important translational step in the identification of ASIC1a as a potential therapeutic target for stroke.

## Introduction

Globally, stroke is the second leading cause of death and the third leading cause of disability. Moreover, the incidence of stroke is rapidly growing (Feigin et al., 2021). Projections show that by 2030, approximately 4% of US adults will have had a stroke (Tsao et al., 2022). Among patients recovering from stroke, age-related functional decline is accelerated, with the annual increase in disability increasing considerably after stroke. A year after the stroke, survivors report increased dependence on caregivers for daily living and activities like toileting, walking, transportation, shopping, and laundry (Tsao et al., 2022).

Ischemic stroke is the most common type of stroke and occurs when there is a loss of blood circulation to an area of the brain. The only FDA-approved therapeutic for the treatment of ischemic stroke is tissue plasminogen activator (tPA). The number of patients who can be treated with tPA is limited as there is a 4.5 h time constraint from the onset of symptoms for administration. Unfortunately, even when tPA is administered within the 4.5 h time limit, there is a fourfold increased risk of intracerebral hemorrhage (leading to an almost 50% mortality) when compared with patients given a placebo. This, along with the continued failure of clinical trials testing various therapeutics (e.g., glutamate antagonists), raises the need for continued study of brain injury mechanisms to unveil novel therapeutic strategies for ischemic stroke.

Brain acidosis occurs after ischemia due to an increase in anaerobic glycolysis leading to an accumulation of lactic acid. Brain pH is also decreased by the release of protons from the presynaptic terminal and ATP hydrolysis (Nedergaard et al., 1991). During ischemia, extracellular pH commonly falls to 6.5 and in severe instances can get lower than 6.0. It has been well established that acidosis exacerbates ischemic brain damage; however, the detailed mechanism remains unknown. Acid-sensing ion channels (ASICs) are a family of cation channels that are activated by acidosis (Krishtal and Pidoplichko, 1980, 1981). ASIC1a channels are permeable to both Na^+^ and Ca^2+^ and mediate an inward current in response to acidic extracellular pH resulting in membrane depolarization. Previous work has shown that the ischemic injury caused by acidosis is mediated by activation of Ca^2+^-permeable ASICs, independent of glutamate receptors and voltage-gated Ca^2+^ channels (Xiong et al., 2004). Additionally, it has been shown that ASIC1a inhibition has a ∼5 h therapeutic time window (Pignataro et al., 2007). This work has presented substantial evidence that ASICs play an important role in ischemic brain injury, providing a novel therapeutic target for stroke intervention. However, this work has mostly focused on the short-term effects of ASIC1a inhibition. The goal of the current work is to determine if deletion or inhibition of ASIC1a results in long-term behavioral improvements after stroke.

## Materials and Methods

### Animals

Male wild-type (ASIC1a^+/+^) and ASIC1a knock-out (ASIC1a^−/−^) mice with congenic C57BL/6 background aged 2–4 months were used to assess behavioral and histological outcomes 1–28 d following the ischemic stroke induction. The animals were single housed with standard food and water *ad libitum* on a 12 h light/dark cycle. All animal procedures were reviewed and approved by the Institutional Animal Care and Use Committee (IACUC) of Morehouse School of Medicine.

### Stroke induction

Transient focal ischemia was induced by suture occlusion of the middle cerebral artery (MCAO) for 30–60 min, as described in previous studies (Xiong et al., 2004). Animals were anesthetized using 1.5% isoflurane, 70% N_2_O, and 28.5% O_2_ with intubation and ventilation. Rectal and temporalis muscle temperatures were maintained at 37°C ± 0.5°C with a thermostatically controlled heating pad and lamp. Cerebral blood flow was monitored by transcranial laser Doppler. Animals with blood flow above 20% during ischemia were excluded (Xiong et al., 2004). Sham animals were subjected to the same procedure, but the suture was not tightened around the middle cerebral artery.

### Intranasal psalmotoxin 1 administration

ASIC1a^+/+^ mice were assigned to treatment groups in a blinded fashion. All mice received a single dose of either vehicle (lactated ringers; 50 µl) or psalmotoxin 1 (PcTX1) solution (50 µl, 1 µM) administered intranasally 30 min after MCAO. A total volume of 50 μl was delivered with an Eppendorf pipette in 10–12 μl drops to alternating nostrils. Individuals blinded to treatment groups performed all manipulations and analyses.

### Mortality and weight

Mortality was classified as death occurring in the acute (2–48 h post-MCAO), subacute (3–7 d post-MCAO), or chronic (>7 d post-MCAO) phase of stroke recovery. Animals in both groups were provided soft food and injected subcutaneously with 1 ml of lactated ringers for 3 d following MCAO. Body weight (BW) was measured prior to surgery (i.e., baseline) and daily thereafter until day 7 and then measured weekly.

### Infarct quantification

Twenty-four hours after MCAO, animals were killed, and brains were sectioned coronally at 1 mm intervals and stained by vital dye immersion with 2% 2,3,5-triphenyltetrazolium hydrochloride (TTC) in paraformaldehyde solution. Infarction area was calculated by subtracting the area stained with TTC in the ischemic hemisphere from the area of the nonischemic hemisphere. Surgery and analyses were performed by individuals blinded to experimental groups.

### Behavioral tests

All tests were performed by the same experimenter that was blinded to the genotype and treatment group of mice from postoperative day 1–28 between 12:00 h and 14:00 h. All animals were evaluated before ischemia and then on postischemic days 1, 3, 5, 7, 14, 21, and 28. The order of test descriptions below corresponds to the chronological order that tests were performed on test days. The neurological deficit tests were performed and scored in real time. The open field tests were video-recorded and then automatically scored using Noldus EthoVision XT 14 (Noldus IT, The Netherlands). The corner test was video-recorded and then scored manually.

### Neurological deficit

Neurobehavioral deficit was assessed using a modified version of the methods described by Lourbopoulos et al. to score both focal neurological deficits and systemic general deficits (Lourbopoulos et al., 2017). Focal deficits, functional outcomes that are directly related to the brain injury and affect a specific region/part of the body, were evaluated by body symmetry, gait, circling behavior, forelimb symmetry, hindlimb symmetry, compulsory circling, whisker response, and trunk. General deficits, systemic processes that are affected by stroke severity but not specific to a certain area of the brain, were evaluated by examining hair, ears, eyes, spontaneous activity, and anxiety behavior. These tests were chosen as a quick and simple assessment of neurological deficit after stroke.

### Open field

To evaluate whether the presence of ASIC1a affects mobility behaviors after stroke, locomotion was recorded using an open field test via an electronic imaging system (Noldus EthoVision XT). The arena contained two circular zones: a center (diameter, 30 cm) and a peripheral zone next to the wall (diameter, 55 cm). Mice were placed in the center of the arena and allowed to explore for 4 min. The results of the open field test were expressed as total distance moved and time in zones.

### Corner test

To evaluate differences in sidedness bias after MCAO, mice were placed in a corner with a 30° angle. A small opening was cut into the corner to motivate the animals to explore the corner. Once placed, the mice were left to explore the corner freely. After entering the corner, animals typically rear and turn to the left or right side (Zhang et al., 2002; Balkaya et al., 2013). There was no time limit to record the moves. The first five successful turns after approaching the corner were recorded and noted as “right turn” or “left turn.” The directional results of the corner test were expressed as % of the total number of turns.

### Statistical analysis

Survival data are represented by the percentage surviving and were analyzed via log rank test. All other values are expressed as means ± SEM, and statistical analysis was performed via *t* tests or ANOVA followed by the Dunnett's test. Statistical significance was accepted at the 95% confidence level (*p* < 0.05).

## Results

### Knock-out of ASIC1a did not impact mortality or weight after 30 min MCAO

To determine if knock-out of ASIC1a impacts mortality and weight after 30 min MCAO, ASIC1a^+/+^ and ASIC1a^−/−^ mice were monitored for 28 d following MCAO. A total of 10 ASIC1a^+/+^ mice were used for the survival experiment; one animal was killed during surgery due to complications, so it is excluded from this analysis. 89% of ASIC1a^+/+^ mice survived the first 24 h after MCAO and 67% survived the entire 28 d of the experiment. A total of 11 ASIC1a^−/−^ mice were used for the survival experiment. In addition, 100% of ASIC1a^−/−^ mice survived the first 24 h after MCAO, and 73% survived the entire 28 d of the experiment. There was no significant difference in survival between ASIC1a^+/+^ and ASIC1a^−/−^ mice after 30 min MCAO.

**Figure 1.**
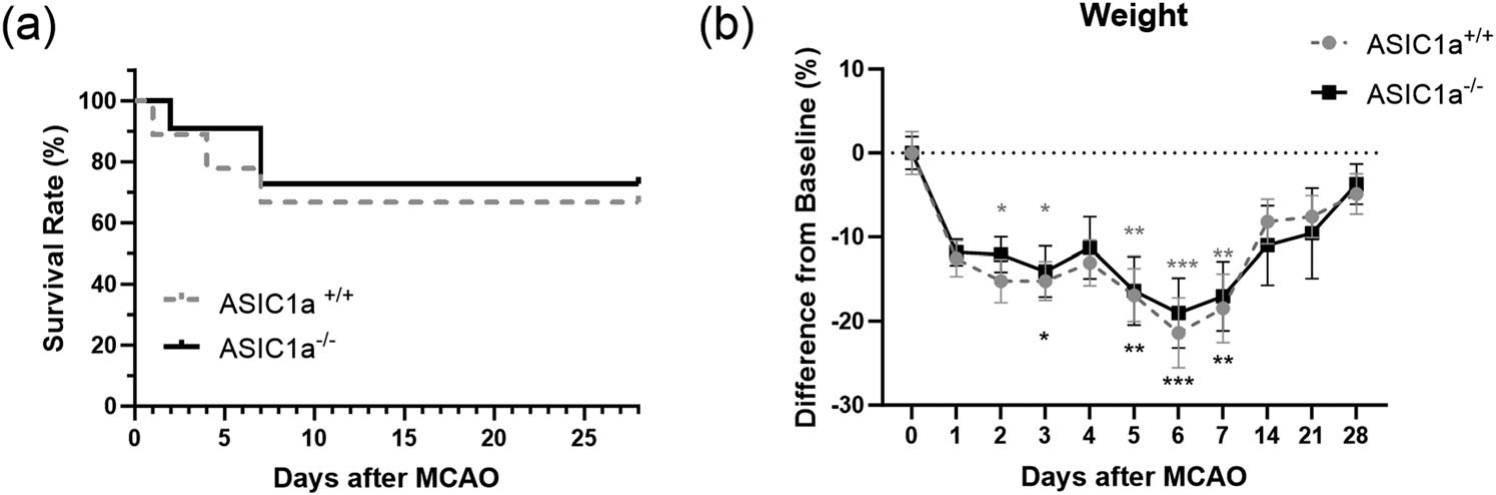
ASIC1a deletion does not impact mortality or weight after MCAO. ***a***, ASIC1a^+/+^ and ASIC1a^−/−^ mice did not significantly differ in survival for up to 28 d after MCAO. Survival data are presented as percentage surviving and analyzed by log rank test. ***b***, ASIC1a^+/+^ and ASIC1a^−/−^ mice also did not have significant differences in weight changes for up to 28 d after MCAO. Weight data are presented as mean ± SEM and analyzed by ANOVA (*n* = 9–11 for each group). The asterisks represent the significant changes in weight as compared with baseline measurement. *p < 0.05, **p < 0.01, ***p < 0.001.

To determine if there were differences in weight loss and gain between ASIC1a^+/+^ and ASIC1a^−/−^ mice, all animals were weighed on the day of surgery, daily for the first 7 d post-MCAO, and then weekly thereafter. There were no significant differences in weight loss or gain between ASIC1^+/+^ and ASIC1a^−/−^ mice when compared with each other on any day post-MCAO; however, there was some differences in the timing of weight loss when compared with baseline weight post-MCAO. ASIC1a^+/+^ and ASIC1a^−/−^ mice had a similar drop in weight 1 d after MCAO (−12.6 ± 2.1 and −11.8 ± 1.6 percent difference from baseline, respectively). ASIC1a^+/+^ mice had a quicker weight loss, with weight becoming significantly different from baseline starting at day 2 post-MCAO (−15.1 ± 2.6, *p* = 0.01; Fig. 1*b*). The weight of ASIC1a^−/−^ mice did not become significantly different from baseline until day 3 post-MCAO (−14.09 ± 3.0, *p* = 0.02). Both ASIC1a^+/+^ and ASIC1a^−/−^ mice hit their lowest weight on day 6 (−21.4 ± 4.2 and −19.0 ± 4.2, respectively) post-MCAO. ASIC1a^+/+^ and ASIC1a^−/−^ mice also both began regaining weight on day 7, ASIC1a^+/+^ mice going from a −21.4% weight loss from baseline on day 6 to a −18.5% weight loss on day 7, and ASIC1a^−/−^ mice going from a −19.0% weight loss from baseline on day 6 to a −17.0% weight loss on day 7. By day 28 post-MCAO, both ASIC1a^+/+^ and ASIC1a^−/−^ mice had regained most of their baseline weight (−4.8 ± 2.4 and −3.7 ± 2.4, respectively).

### Reduced infarct volume in ASIC1a^−/−^ mice

**Figure 2.**
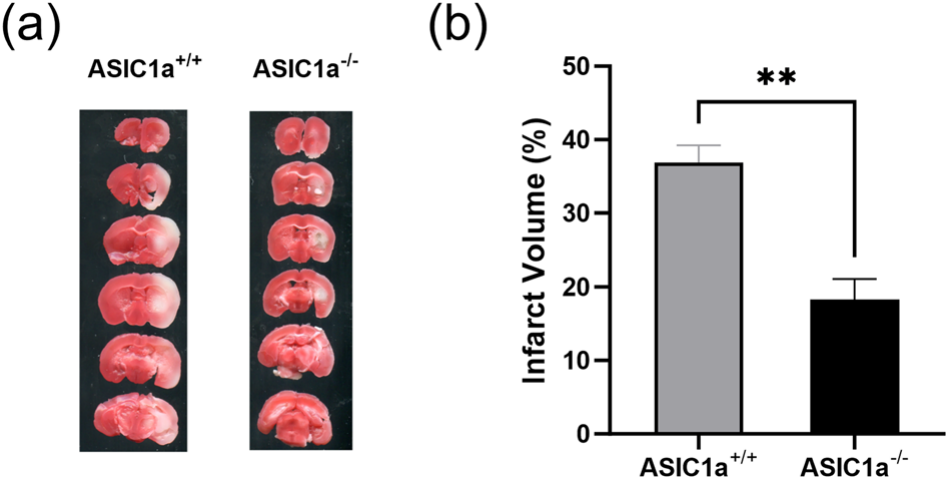
Protective effect of ASIC1a knock-out on infarct volume after MCAO. ***a***, Twenty-four hours after 30 min MCAO, mice were killed, and infarct volumes were determined using TTC staining. ***b***, Knock-out of ASIC1a significantly reduced brain infarct volume. Data are presented as mean ± SEM and analyzed by *t* test (*n* = 4–5 for each group). *p < 0.05 when compared with ASIC1a^+/+^.

It has been shown previously that ASIC1a knock-out reduced the infarct volume after 60 min MCAO (Xiong et al., 2004). To determine if infarct volume is also reduced in ASIC1a^−/−^ mice after 30 min MCAO, brains were removed at 24 h post-MCAO and stained with TTC. As expected, ASIC1a^−/−^ mice had significantly reduced infarct volume (18.3 ± 2.9%) when compared with ASIC1a^+/+^ mice (36.9 ± 2.3%, **p < 0.01; Fig. 2).

### Neurological functions recovered more rapidly in ASIC1a^−/−^ mice

**Figure 3.**
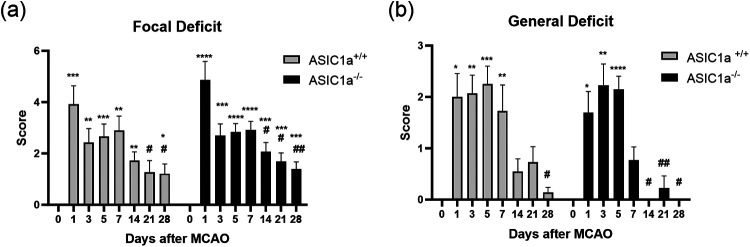
Knock-out of ASIC1a accelerates neurological deficit recovery. Neurological scoring was done prior to MCAO for baseline measurement and at the specified timepoints post-MCAO. Both ASIC1a^+/+^ and ASIC1a^−/−^ mice had significant focal deficits over a 28 d period following MCAO. ***a***, The focal deficit of ASIC1a^−/−^ mice significantly improved from day 1 deficit a week earlier than ASIC1a^+/+^ mice. ***b***, ASIC1a^−/−^ mice had general deficits for fewer days and significantly improved from day 1 deficit 14 d earlier than ASIC1a^+/+^ mice. Data are presented as mean ± SEM and analyzed by two-way ANOVA (*n* = 11–13 for each group). The asterisks represent the significant differences as compared with day 0 measurement. *p < 0.05, **p < 0.01, ***p < 0.001, ****p < 0.0001. The pound signs represent the significant differences as compared with day 1 measurement. ^#^*p* < 0.05, ^##^*p* < 0.01, ^###^*p* < 0.001, ^####^*p* < 0.0001.

To evaluate the neurological deficits following MCAO, we used a stroke scale that was previously validated to measure focal and general deficits (Lourbopoulos et al., 2017). There were no significant differences in focal or general neurological deficits in ASIC1a^+/+^ versus ASIC1a^−/−^ mice at any of the timepoints post-MCAO. However, there were differences in the speed of recovery between ASIC1a^+/+^ and ASIC1a^−/−^ mice when post-MCAO day 1 deficit scores were compared with the following recovery days. We found that both ASIC1a^+/+^ and ASIC1a^−/−^ mice had focal deficits that lasted for 28 d. However, ASIC1a^−/−^ mice significantly recovered from day 1 post-MCAO focal deficit 7 d sooner than ASIC1a^+/+^ mice (*p* < 0.05 at day 14 and *p* < 0.05 at day 21, respectively; Fig. 3*a*). We found that ASIC1a^+/+^ mice had general deficits that lasted for 7 d and ASIC1a^−/−^ mice had general deficits that lasted for 5 d. Additionally, ASIC1a^−/−^ mice significantly recovered 14 d sooner than ASIC1a^+/+^ mice (*p* < 0.05 at day 14 and *p* < 0.05 at day 28, respectively) when general deficit at day 1 post-MCAO was compared with that of later recovery days (Fig. 3*b*).

### Mobility recovery is improved in ASIC1a^−/−^ mice

**Figure 4.**
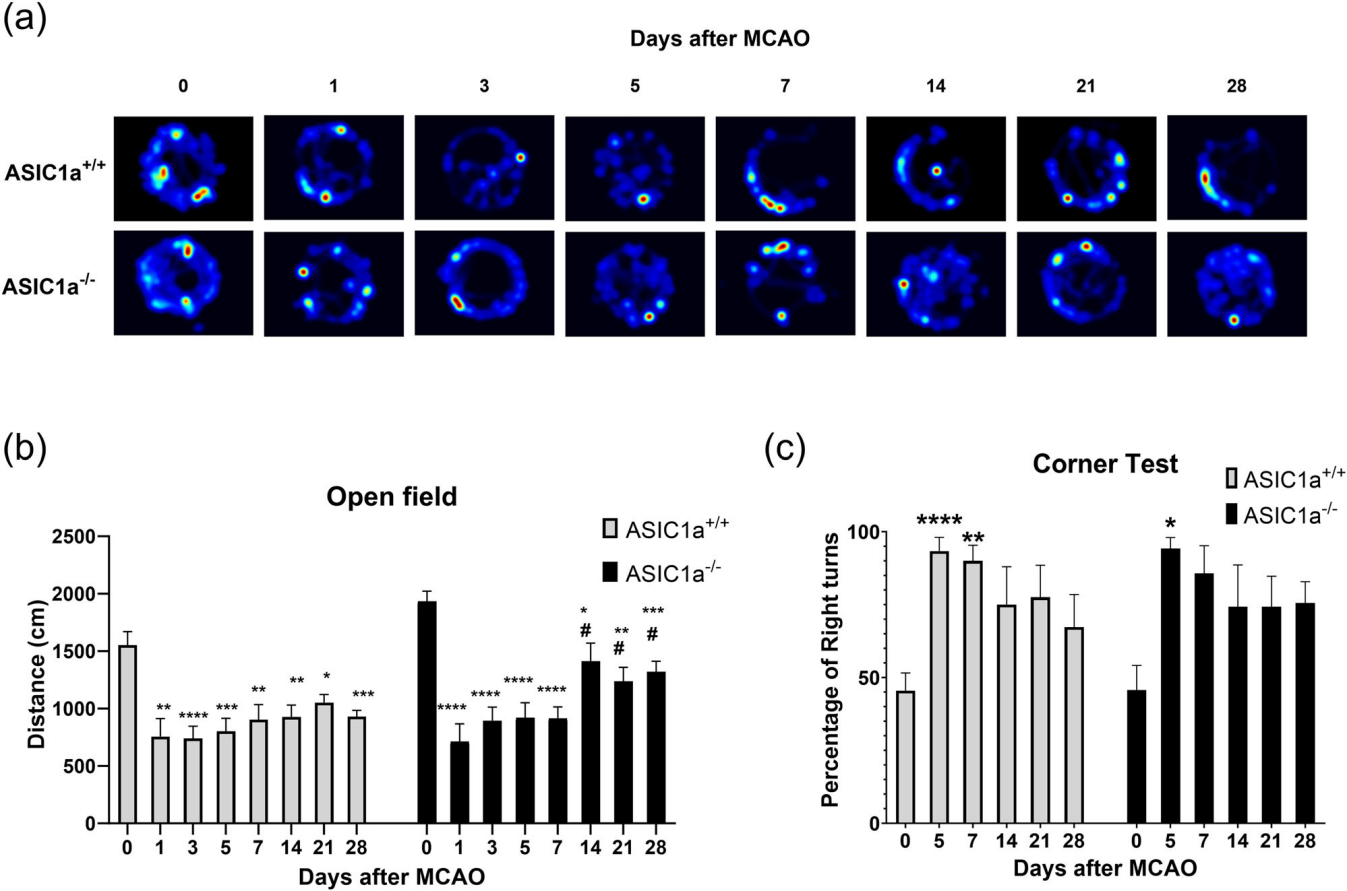
Knock-out of ASIC1a improved mobility recovery but not sidedness bias after stroke. ***a***, Representative visualization of the open field test. ***b***, Both ASIC1a^+/+^ and ASIC1a^−/−^ mice had reduced mobility after MCAO. Only ASIC1a^−/−^ mice significantly recovered mobility. ***c***, Both ASIC1a^+/+^ and ASIC1a^−/−^ mice had significantly right-sidedness bias after MCAO and neither group significantly recovered within 28 d. Data are presented as mean ± SEM and analyzed by two-way ANOVA (open field *n* = 13–18 for each group, corner *n* = 7–11 for each group). The asterisks represent the significant differences as compared with baseline measurement. *p < 0.05, **p < 0.01, ***p < 0.001, ****p < 0.0001. The pound signs represent the significant differences as compared with first day of measurement after MCAO. ^#^*p* < 0.05, ^##^*p* < 0.01, ^###^*p* < 0.001, ^####^*p* < 0.0001.

To evaluate differences in mobility after 30 min MCAO, we used the open field test. At baseline ASIC1a^−/−^ mice were more mobile than ASIC1a^+/+^ mice, but there were no significant differences in mobility between the two groups on any post-MCAO day. Mobility was significantly decreased from baseline at 1 d post-MCAO and remained significantly lower for the entire 28 d for both ASIC1a^+/+^ and ASIC1a^−/−^ mice. However, the rate at which mobility was recovered after MCAO differed between ASIC1a^+/+^ and ASIC1a^−/−^ mice. ASIC1a^−/−^ mice had significantly improved mobility from day 1 post-MCAO to post-MCAO day 14, and that improvement was maintained on days 21 and 28 (*p* < 0.05; Fig. 4*a,b*). In contrast, the mobility in ASIC1a^+/+^ mice did not improve significantly from 1 d post-MCAO.

### Corner turn test

To evaluate differences in sidedness bias after MCAO, we used the corner test. Both ASIC1a^+/+^ and ASIC1a^−/−^ mice significantly increased their preference for the right side on day 5 post-MCAO. Interestingly, ASIC1a^+/+^ mice were significantly more biased toward the right side on days 5 and 7 post-MCAO (*p* < 0.0001 and *p* < 0.005, respectively), while ASIC1a^−/−^ mice were significantly more biased toward the right side on days 5 and 28 post-MCAO (both *p* < 0.05; Fig. 4*c*). Neither ASIC1a^+/+^ nor ASIC1a^−/−^ significantly recovered from the right-sidedness bias caused by MCAO during the 28 d of measurement when comparing day 5 post-MCAO bias to the later recovery days.

### Acute inhibition of ASIC1a using PcTX1 reduced infarct volume after MCAO

**Figure 5.**
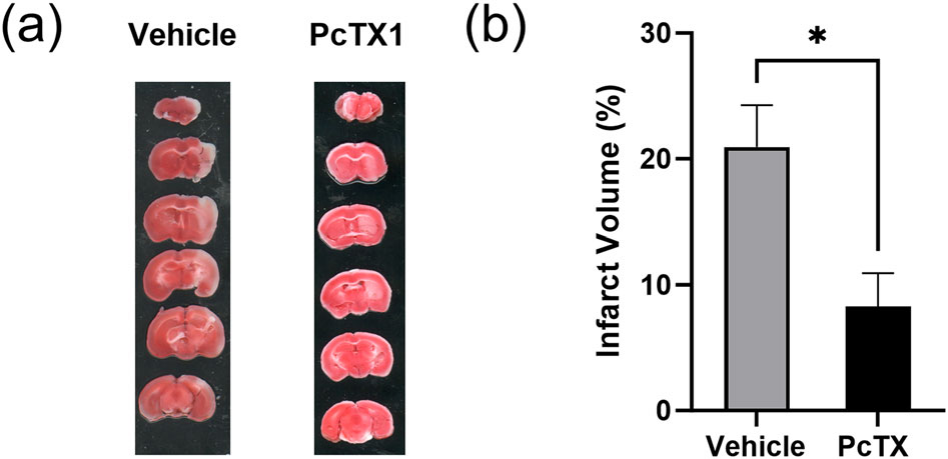
Protective effect of ASIC1a inhibition on infarct volume after focal cerebral ischemia. ***a***, Representative images of TTC stained brains 24 h after 30 min MCAO. ***b***, PcTX1-treated mice had a significantly reduced infarct volume when compared with vehicle mice. Data are presented as mean ± SEM and analyzed by *t* test (*n* = 5 for each group). *p < 0.05 when compared with vehicle.

To determine if acute inhibition of ASIC1a would also result in improved behavioral recovery, we administered PcTX1 intranasally to wild-type mice after 30 min MCAO. PcTX1 was administered at 30 min post-MCAO, and brains were removed at 24 h post-MCAO and stained with TTC. As expected, mice given PcTX1 had significantly reduced infarct volume (8.28 ± 2.7%) when compared with vehicle mice (20.94 ± 3.3%, *p* < 0.05; Fig. 5)[Fig eneuro-11-ENEURO.0341-23.2023F6].

**Figure 6. eneuro-11-ENEURO.0341-23.2023F6:**
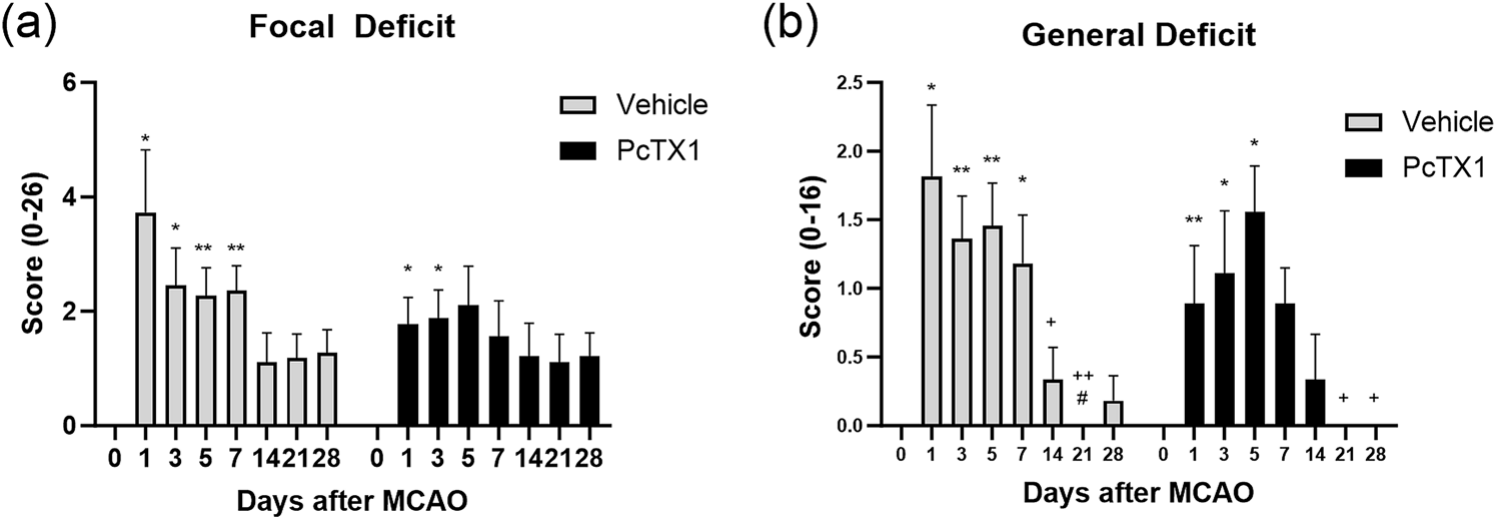
Acute inhibition of ASIC1a improves neurological deficit recovery. Neurological scoring was done prior to MCAO for baseline measurement and at the specified timepoints post-MCAO using the previously described stroke scale. ***a***, PcTX1 mice have significantly increased focal deficits for the first 3 d, and vehicle mice had significantly increased deficits for 7 d post-MCAO. ***b***, PcTX1 mice had significantly increased general sickness on days 1, 3, and 5 post-MCAO and significantly recovered from that increase on day 21 post-MCAO. Vehicle mice had significantly increased general sickness on all days up to 7 d post-MCAO and significantly recovered from that increase on day 21 post-MCAO. Data are presented as mean ± SEM and analyzed by two-way ANOVA (*n* = 9–11 for each group). The asterisks represent the significant differences as compared with baseline measurement *p < 0.05, **p < 0.01, ***p < 0.001, ****p < 0.0001. The pound signs represent the significant differences as compared with day 1 measurement ^#^*p* < 0.05, ^##^*p* < 0.01, ^###^*p* < 0.001, ^####^*p* < 0.0001. The crosses represent the significant differences as compared with day 5 measurement ^+^*p* < 0.05, ^++^*p* < 0.01, ^+++^*p* < 0.001, ^++++^*p* < 0.0001.

### Neurological deficits were reduced after MCAO in PcTX1-treated mice

We evaluated neurological deficits as described above. There were no significant differences in focal or general neurological deficits in vehicle versus PcTX1 mice at any timepoints post-MCAO. However, vehicle mice had a significant increase in focal deficit for 7 d post-MCAO when compared with baseline, whereas PcTX1 mice only had significant focal deficit for 3 d post-MCAO (Fig. 6*a*). Vehicle mice also had a significantly increased general deficit for the first 7 d post-MCAO and began to significantly recover from those deficits on day 14 post-MCAO. PcTX1 mice only had a significant general deficit on days 1,3, and 5 post-MCAO and significantly recovered from those deficits on day 21 post-MCAO (Fig. 6*b*).

### Mobility was slightly impaired after MCAO in PcTX1 mice

**Figure 7.**
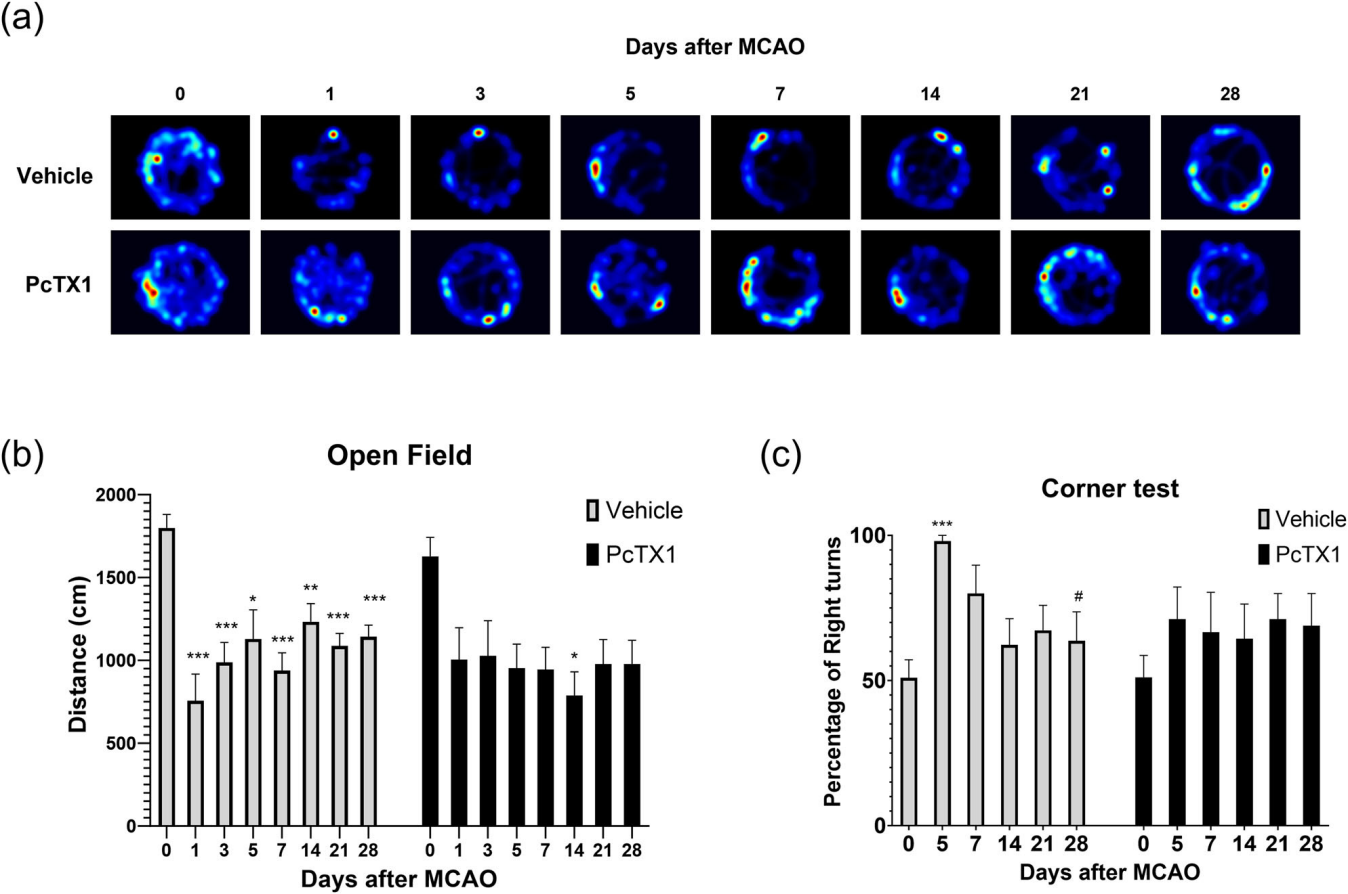
Acute inhibition of ASIC1a improved mobility recovery and sidedness bias after stroke. ***a***, Representative visualization of the open field test. (***b***) Vehicle mice had reduced mobility for all days post-MCAO and did not significantly recover within 28 d post-MCAO. PcTX1 mice only had significantly reduced mobility on day 14 post-MCAO. ***c***, Vehicle mice had severe right-sidedness bias on day 5 post-MCAO and significantly recovered by day 28 post-MCAO. PcTX1 mice did not have a significant increase in sidedness bias. Data are presented as mean ± SEM and analyzed by two-way ANOVA (*n* = 11–9 for each group). The asterisks represent the significant differences as compared with baseline measurement. *p < 0.05, **p < 0.01, ***p < 0.001, ****p < 0.0001. The pound signs represent significant differences as compared with day 1 measurement. ^#^<≤0.05, ^##^*p* < 0.01, ^###^*p* < 0.001, ^####^*p* < 0.0001.

To evaluate differences in mobility after MCAO, we used the open field test as described above. Vehicle mice had significantly impaired mobility when compared with baseline on all days post-MCAO, with no significant improvement. PcTX1 mice only incurred a significant decrease in mobility on 1 d (day 14) post-MCAO (Fig. 7*a,b*).

We evaluated differences in sidedness bias recovery in Vehicle and PcTX1 mice as described above. Vehicle mice significantly increased their preference for the right side on day 5 post-MCAO and significantly recovered by day 28 post-MCAO. PcTX1 mice did not incur a significant increase in sidedness bias at all for the full 28 d of measurement post-MCAO (Fig. 7*c*).

## Discussion

Stroke continues to be a leading cause of death and disability worldwide, despite extensive research efforts. The failure to translate preclinical treatments to clinical practice in humans is often attributed to the lack of similarities between preclinical and clinical research endpoints (Endres et al., 2008; Gannon, 2014; Lourbopoulos et al., 2021). One factor that may contribute to the eventual clinical failure of stroke treatments that were effective in preclinical studies is the focus on acute molecular or histological endpoints like infarct size rather than clinically relevant endpoints. In clinical studies for stroke, the core target endpoint is clinical improvement which is measured as clinical neurological improvement at later time points using the modified Rankin scale (Broderick et al., 2017). Additionally, since much of the focus of preclinical trials is on differences in infarct volume (measured within 24–72 h), improvements in long-term behavioral outcomes are rarely considered as the primary endpoint. The typical clinical stroke patient is monitored for neurological improvement for weeks or months following the stroke, rather than hours or days. Consequently, there is an increasing need to assess long-term functional and behavioral outcomes in preclinical research to reduce the “translational roadblock” to the development of stroke therapies. The contribution of ASICs to neuronal injury during the acute phase of stroke is well studied. The current work sought to bridge the translational gap by investigating the effect of ASIC1a on long-term behavioral outcomes after stroke.

Neurological scoring was developed for human clinical trials to measure patient's performance in activities of daily life (D'Olhaberriague et al., 1996). The most widely used neurological scoring scale is the modified Rankin scale because of its sensitivity, validity, simplicity of use, and interpretability (Wolfe et al., 1991; de Haan et al., 1995; Banks and Marotta, 2007). However, there are various scoring systems that have been validated in humans including the stroke impact scale and the Barthel index (Duncan et al., 1999). Rodents and humans exhibit multiple similarities in sensorimotor and neurological deficits after stroke (Grabowski et al., 1993; Zausinger et al., 2000). However, due to anatomical and functional differences between rodents and humans, accurate assessment of neurological function after stroke is dependent on the use of appropriate behavioral tests. Like in humans, several scoring systems have also been developed to evaluate stroke outcomes in rodents. We used a modified version of a previously validated experimental stroke scale (ESS) that was developed by Lourbopoulos et al. (2017). The ESS includes two components, a focal component that evaluates focal neurological deficits and a general component that evaluates systemic behavioral deficits. These two components are comparable to clinical measurements of weakness/degree of disability and ability to perform activities of daily living (ADLs), respectively (Narayanaswami, 2017).

The primary and secondary endpoints in ischemic stroke clinical trials are often measured as improvements in modified Rankin score over time. We chose to evaluate recovery in our behavioral tests with a similar approach. First, we analyzed the impact of the MCAO by comparing the baseline measurement to the measurements taken after MCAO during recovery. Then, we analyzed the improvement post-MCAO by comparing day 1 post-MCAO measurements to the rest of the recovery days. At baseline, ASIC1a^+/+^ and ASIC1a^−/−^ mice performed similarly in most behavioral tests, except for the open field test where ASIC1a^−/−^ mice are more active at baseline. This is consistent with a previous study, which demonstrated that ASIC1a^−/−^ mice show fewer signs of fear and anxiety in the open field (Coryell et al., 2007). Like vehicle-treated ASIC1a^+/+^ mice, mice that had ASIC1a either knocked out or pharmacologically inhibited showed significant focal and general neurological deficits following stroke. However, ASIC1a knocked out or inhibited mice showed improvement in both the focal and general neurological scores more quickly than vehicle-treated ASIC1a^+/+^ mice. This pattern of similar initial impact on both groups but increased speed of recovery among ASIC1a^−/−^ mice was also shown in the open field test, where ASIC1a^−/−^ mice displayed significant improvement in mobility by day 14, while ASIC1a^+/+^ mice did not show significant improvement for the duration of the study. The pattern of increased speed of recovery did not show in the corner test; however, this could be a limitation of the 28-day timescale used, as neither ASIC1a^+/+^ nor ASIC1a^−/−^ mice significantly recovered within the 28 d of testing. Mice that had ASIC1a acutely inhibited via PcTX1 did not incur significant impairments in mobility for most of the test days. Additionally, PcTX1-treated mice did not incur any significant increase in right-sidedness bias for the duration of the experiment, likely due to the smaller infarct size.

Inflammation plays a key role in the pathogenesis of stroke and has become a target for therapeutic intervention. However, inflammatory cells are multifunctional, playing both beneficial and detrimental roles where inhibiting a pathway at the wrong time could lead to exaggerated pathogenesis (Jayaraj et al., 2019). The CNS response to ischemic insult happens in three broadly defined overlapping phases: (1) cell death and inflammation, (2) cell proliferation for tissue replacement, and (3) tissue remodeling (Burda and Sofroniew, 2014). The first phase, cell death and inflammation, begins rapidly over a time scale of seconds to hours, with more gradually progressing events developing over days. Brain acidosis occurs during the early parts of this phase, causing the activation of ASICs leading to cell death. The dead and dying cells release “danger signals” that activate the innate immune response. There has been evidence suggesting that ASIC1a mediates these “danger signals” by activating voltage-activated K^+^ channels which in turn activate inflammasomes (Rajamäki et al., 2013; Wang et al., 2015). Inflammasomes are an assembly of pattern-recognition receptors (PRRs) that control the maturation and secretion of potent proinflammatory interleukins (IL-1β and IL-18). The inflammation process in the first phase of stroke recovery is intrinsically bound to the processes that follow in the second and third phases which mark the beginning of recovery via cell proliferation for tissue replacement and tissue remodeling. There is little literature on the role of ASIC1a in inflammatory cascades, and that is especially true for long-term mechanisms. Thus, the role that ASIC1a plays in long-term stroke recovery remains unclear. The exact reason why PcTX1-injected mice performed better after stroke than the ASIC1a^−/−^ mice is unclear. One possibility is that normal ASIC1a function might play a role in the recovery phase of the stroke. PcTX1 applied in the acute phase of stroke reduces the infarct volume without interfering with the potential beneficial function of the channel in the recovery phases. In contrast, although knock-out of the channel can reduce the infarct volume in the acute phase, it also eliminates the potential beneficial function of the channel in the recovery phases. Future work focusing on elucidating the role of ASIC1a during poststroke recovery phases could help provide better explanations.

Our study had some limitations. In this study, we used 30 min MCAO. ASIC1a inhibition may be less protective in circumstances where more severe damage is caused by a 60 min or longer MCAO. Also, the ASIC1a^−/−^ mice used were full-body genetic knockouts, and as such they could give insight into the global role of ASIC1a in long-term stroke recovery. However, this limited our ability to draw brain mechanism-specific conclusions on its role in behavioral recovery. Another limitation of our study is that PcTX1 was administered once at 30 min post-MCAO. Acute cell death after ischemic stroke can last for up to 48 h. Administration of PcTX1 for at least the first 48 h may reduce the negative impact of stroke on behavior even more. Additionally, administering PcTX1 so quickly after MCAO is not a realistic representation of how an ASIC1a inhibitor would be administered in a clinical setting. Pignataro et al. (2007) found that intranasal administration of PcTX1 was effective at reducing infarct size even when administered up to 5 h after MCAO. Future work will apply PcTX1 at later timepoints that are more typical for a clinical setting.

The major goal of this study was to determine if inhibition of ASIC1a channels would result in improved long-term behavioral recovery after stroke. The contribution of ASIC1a to neuronal injury during the acute phase of stroke is well studied, and we sought to bridge part of the translational gap by focusing on long-term behavior recovery. We show for the first time in an in vivo model that both genetic knock-out and acute inhibition of ASIC1a channels improve long-term recovery after stroke. This study expands on previous work by demonstrating that the reduction in tissue damage caused by inhibiting ASIC1a translates into significant improvements in speed of recovery in focal deficits, general deficits, and mobility. This study also supports and extends previous work demonstrating ASIC1a as a potential therapeutic target for the treatment of ischemic stroke.

## Synthesis

Reviewing Editor: Arianna Maffei, Stony Brook University Decisions are customarily a result of the Reviewing Editor and the peer reviewers coming together and discussing their recommendations until a consensus is reached. When revisions are invited, a fact-based synthesis statement explaining their decision and outlining what is needed to prepare a revision will be listed below. The following reviewer(s) agreed to reveal their identity: Quanguang Zhang, Carina Weissman.

The reviewer agree that the manuscript is interesting and overall well-written.

They both asked minor changes to the text and legends to clarify symbols and increase readability.

The specific points raised by the reviewers are listed below.

Reviewer #1

The role of acid-sensing ion channel 1a (ASIC1a) in brain injury has gained increasing research interest recently. Using both ASIC1a inhibitor PcTX1 and ASIC1a knockout animal models, the manuscript showed that inhibition or depletion of ASIC1a remarkably improved neurological deficits and mobility after brain ischemia. These findings provided valuable insights into the contribution of ASIC1a to functional deficits after stroke and would be of great interest to this field.

Some minor comments to improve the manuscript are as follows.

• "The behaviors of mice were evaluated before and after the stroke ..." The term "behaviors" could be more specific and accurate (e.g., neurological consequences).

•For intranasal psalmotoxin 1 administration, did all animals (both mice sacrificed 24 hours after MCAO and those sacrificed 28 days after MCAO) receive a single dose of intranasal Psalmotoxin 1 administration? It would be helpful to clarify it.

• The concept of "focal deficits" and "general deficits" (Figure 3 and 6)could be explained in more detail. What does the "#" symbol signify in Figure 4?

•Although the manuscript is well prepared. Some sentences could be more concise and clear, as follows:

- however, ASIC1a-/- mice (p<0.05 on days 14, 21, and 28) significantly recovered 14 days sooner than ASIC1a+/+ mice (p<0.05 at day 28) when general deficit at day 1 post-MCAO is compared to later recovery days.

- ASIC1a-/- mice had general deficits for fewer days and significantly improved from day 1 deficit 14 days earlie than ASIC1a+/+ mice. (typo: earlie).

- Spellings: sometime use "PcTX", better to be read consistent.

• The authors could explain the observed higher mobility of ASIC1a-/- mice compared to ASIC1a+/+ mice at baseline. Does it expect to have an impact on the interpretation of the study results？

•Figure 3, "Both ASIC1a+/+ and ASIC1a-/- mice had significant focal deficits for the full 28 days following MCAO". The neurological scoring was conducted on days 1, 3, 5, 7, 14, 21, and 28 post-MCAO, rather than full 28 days. It would be helpful to use 'over a 28-day period' instead.

Reviewer #2

Minor comments:

- Minor Comments:

Figure 4: The legend is missing an explanation for the "#" symbol.

In the same Figure, the X-y axis uses a different character type, and the spacing for the X-axis should be increased.

References: The publication by Xiong, Z.-G., Zhu, X.-M., Chu, X.-P., Minami, M., Hey, J., Wei, W.-L., . . . Simon, R. P. (2004) is listed twice.

In the Discussion section, the authors have carefully considered their findings, compared them with existing studies, and discussed the study's limitations. It might be beneficial to also mention that the previous study (Pignataro et al., 2007) found intranasal administration of Pctx-1 to be effective, especially in reducing infarction volume, even when administered at later time points.

Additionally, it could be valuable to include a discussion of the greater efficacy of Pctx administration compared to the results obtained from knockout animal experiments.
